# Nonlinear ionizing radiation-induced changes in eye lens cell proliferation, cyclin D1 expression and lens shape

**DOI:** 10.1098/rsob.150011

**Published:** 2015-04-29

**Authors:** Ewa Markiewicz, Stephen Barnard, Jackie Haines, Margaret Coster, Orry van Geel, Weiju Wu, Shane Richards, Elizabeth Ainsbury, Kai Rothkamm, Simon Bouffler, Roy A. Quinlan

**Affiliations:** 1 School of Biological and Biomedical Sciences, University of Durham, Durham DH1 3LE, UK; 2 Biophysical Sciences Institute, University of Durham, Durham DH1 3LE, UK; 3 Public Health England, Centre for Radiation, Chemical & Environmental Hazards, Chilton, Didcot, Oxon OX11 0RQ, UK; 4 Faculty of Science, KU Leuven, Kasteelpark Arenberg 11, Leuven 3001, Belgium

**Keywords:** ionizing radiation, eye lens, cyclin D1, γH2AX, DNA DSB, non-linear effect

## Abstract

Elevated cataract risk after radiation exposure was established soon after the discovery of X-rays in 1895. Today, increased cataract incidence among medical imaging practitioners and after nuclear incidents has highlighted how little is still understood about the biological responses of the lens to low-dose ionizing radiation (IR). Here, we show for the first time that in mice, lens epithelial cells (LECs) in the peripheral region repair DNA double strand breaks (DSB) after exposure to 20 and 100 mGy more slowly compared with circulating blood lymphocytes, as demonstrated by counts of γH2AX foci in cell nuclei. LECs in the central region repaired DSBs faster than either LECs in the lens periphery or lymphocytes. Although DSB markers (γH2AX, 53BP1 and RAD51) in both lens regions showed linear dose responses at the 1 h timepoint, nonlinear responses were observed in lenses for EdU (5-ethynyl-2′-deoxy-uridine) incorporation, cyclin D1 staining and cell density after 24 h at 100 and 250 mGy. After 10 months, the lens aspect ratio was also altered, an indicator of the consequences of the altered cell proliferation and cell density changes. A best-fit model demonstrated a dose-response peak at 500 mGy. These data identify specific nonlinear biological responses to low (less than 1000 mGy) dose IR-induced DNA damage in the lens epithelium.

## 2. Introduction

Vision is one of the most important senses to animals, which has evolved successfully to allow spatial definition [1]. In mammals, this sense has been optimized to include, for instance, reduced optical aberrations by the presence of lenses with graded indices [2] and the accommodative ability of the lens in humans and other primates [3]. The eye lens is an avascular tissue contained within its own basement membrane and bathed in the eye humours. A single layer of epithelial cells covers the anterior hemisphere of the lens and progeny from these epithelial cells differentiate into fibre cells that comprise the mass of the lens. Epithelial cell proliferation and differentiation to form lens fibre cells are concentrated in the germinative (GZ) and transitional (TZ) zones of the lens epithelium at the lens equator [4,5]. Lens epithelial cells (LECs) differentiate into fibre cells in this ‘peripheral’ region of the epithelium, entering the body of the lens via the meridional rows (MR) in the TZ [6], where the timely, organized formation of fibre cells is regulated by, for instance, aPKCλ [7] and src/ephrin A2 [8]. Such proteins ensure the maintenance of the geometric organization of the fibre cells, which is so important to lens function [3,9]. Changes in cell proliferation translate directly into alterations to lens morphology [7,8,10,11]. This peripheral region and specifically the GZ of the lens is known to be radiosensitive due to the concentration of proliferating cells located here [12,13].

Since the end of the nineteenth century, the eye lens has been known to be a radiosensitive tissue [14] and the heightened sensitivity of the lens compared with other ocular tissues was reported in 1929 [15]. Studies from the last century had established that dividing epithelial cells in the GZ were critical to ionizing radiation (IR)-induced cataract [16] as preventing the proliferation of these cells was an efficient radioprotection mechanism [17]. Exposure to high doses (15 Gy) also decreased cell density in this region and disrupted cell organization in the GZ and MR [16]. Recently, a large body of epidemiological evidence from atomic-bomb survivors, clean-up workers, healthcare professionals who use X-rays [18–21] and others has led to the proposed new threshold for radiation cataractogenesis of 0.5 Gy. Indeed, the International Commission on Radiological Protection (ICRP) has recently recommended an occupational equivalent dose limit of 0.02 Sv yr^−1^ (averaged over 5 years, with no single year more than 0.05 Sv yr^−1^) to prevent radiation-induced cataracts [22]. This recommendation has now been incorporated into the revised EU Basic Safety Standards (BSS) [23], a mark of the importance to people's health and well-being. There are, however, no dose-response data for low-dose (less than 0.5 Gy) IR sensitivity of the eye lens. It has been suggested that low-dose IR might cause effects in nonlinear proportion with dose in the lens epithelium [24]. There is also uncertainty in the literature about whether cataract is a deterministic or stochastic consequence of (low-dose) IR equivalent dose [18,25,26]. The current recommended annual exposure limits have also been challenged [27,28]. It is therefore a very important scientific and societal goal to establish the biological responses to low-dose IR.

It is well known that IR causes double strand breaks (DSBs) in DNA, either by direct or indirect means. The purpose and sequence of events involved in the initial DNA damage response and the protein complexes involved in repair processes have been extensively researched [29–32]. One of the key players in initiating the repair of DSBs is the histone variant H2AX [33], activating the downstream pathways that are both intricate in DNA context and cell cycle specific in terms of the protein complexes involved [31,34]. The role of modification of H2AX was discovered when IR was used to generate DSBs, which induced the specific phosphorylation of H2AX on serine 139 (S139), to give rise to γH2AX [35]. This is now a well-established marker for DSBs [36,37].

After phosphorylation of S139 in H2AX, the scaffolding protein MDC-1 (mediator of DNA damage checkpoint protein 1) is recruited to help build specific protein complexes needed for the processing of DSBs. One of these is the MRN (Mre11/RAD50/Nbs1) complex, which is critical for the early (less than 1 h) response to DNA DSBs. Formation of this complex allows other repair proteins to bind [32], including BRCA1 (breast cancer 1, early onset) and its partner BARD1 (BRCA1-associated RING domain 1), 53BP1 (tumour suppressor p53-binding protein 1) and RAD51. 53BP1 is a marker for non-homologous end joining (NHEJ) mediated repair, while RAD51 is a recombinase involved in DSB repair by homologous recombination (HR). Interestingly, RAD51 is thought to bind cyclin D1, which can also participate in the repair of DSBs [38,39]. In the lens, cyclin D1 levels correlate with cell proliferation in the GZ at the lens periphery [11,40]. This zone is also believed to be most sensitive to IR damage [12,13]. Compromising the levels of several DNA repair proteins, such as Atm (ataxia telangiectasia mutated) [41] and RAD9 [42] increased the radiosensitivity of the lens. From a radiation protection perspective, radiation cataracts are currently viewed as a threshold effect within the context of a linear-no-threshold interpretation [18,25,26]. It was, however, unknown whether epithelial cells in the lens itself show a linear dose-response by measuring, for instance, markers of DSBs such as γH2AX, 53BP1, RAD51 and cyclin D1.

To address such questions, a low-dose IR exposure model was developed in response to recent ICRP recommendations [22] using mice exposed to 20 mGy–2 Gy X-rays and sacrificed after 1, 3 or 24 h or 10 months post-irradiation. This was a ‘pilot’ study with the key aim of identifying appropriate study methods for low-dose dose-responses in early lens changes, although the 10 month time point also allowed effects on lens morphology to be studied. The results of this study strongly suggest that the eye lens is correctly identified as a radiosensitive tissue, but the data also suggest differential responses dependent upon both IR dose and the location of the epithelial cells within the lens epithelium. Specifically, we demonstrate that the increased radiosensitivity is associated with unusually slow repair of DNA damage in the peripheral region of the lens. When analysed for expression of γH2AX, RAD51 and 53BP1, the peripheral zone demonstrated linear dose-response, but was significantly more sensitive within the low-dose range than cells in the central region and circulating blood lymphocytes. These differences were furthermore correlated with specific low-dose effects upon cyclin D1 levels, EdU labelling and cell density changes in the lens periphery and finally, after 10 months, alteration to lens shape. These data provide evidence of nonlinear effects in the low-dose range of IR that are lens region specific.

## 3. Material and methods

### 3.1. Animal irradiation studies

**Figure 1.**
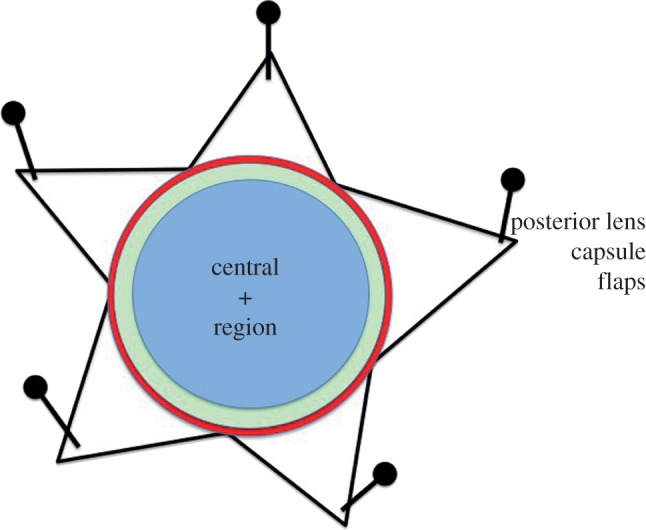
The eye lens and the different regions within the lens epithelium. The lens epithelium can be subdivided into two distinct regions, a central and a peripheral region. The latter comprises two zones called the germinative (GZ) and transitional (TZ) zones. When the anterior lens capsule is flat mounted with the epithelial cells exposed after the removal of the lens fibre mass and the dissected portions of the posterior lens capsule pinned into place, then these regions are apparent. The anterior pole is indicated (+). The central region (blue) is the largest and it is where cell proliferation occurs at a low basal rate. The cells in this region are flatter and less densely spaced. Cell proliferation is largely restricted to the peripheral region and in particular the GZ (green). Proliferating cells were first identified by observing mitotic figures and their incorporation of tritiated thymidine, but now the incorporation of a thymidine analogue such as BrdU or the nucleoside EdU is used. Alternatively, the immunodetection of Ki67, a marker of cells in S-phase, or PCNA is used. Progeny from the GZ cells become lens fibre cells by migrating centripetally towards the lens equator and passing through the TZ and MR (red), before exiting the epithelium via the MR into the body of the lens. MR cells are considered post-mitotic. Cells in the GZ, TZ and MR, comprising the peripheral region of the lens, are shielded from light, but not IR, by the iris and are out of the visual axis.

Six-week-old C57BL/6J mice (Harlan, UK), in groups of two males and two females, were exposed to single doses of IR in an X-ray chamber irradiator (250 kVp, with Gulway generator (AGO Ltd, model no.: CD160/1 Serial no.: 1032–1109; copper- and aluminium-filtered 250kVp X-rays; dose rates of 5 mGy min^−1^ for doses up to 250 mGy and 500 mGy min^−1^ for the 100 and 250, 1000 and 2000 mGy dose points; both dose rates for 100 and 250 mGy). Each animal received a single intraperitoneal injection of EdU (Jena Bioscience GmbH, Germany) at a dose of 90 mg kg^−1^ body weight, 1 h before irradiation. All procedures strictly followed the UK Animals (Scientific Procedures) Act 1986 and had ethical approval of the UK Home Office and local AWERB (Animal Welfare and Ethical Review Body) Committee. Animals were returned to their home cages following X-irradiation for the duration of the experiment and were provided with standard maintenance diet and water ad libitum. For short-term effects, the doses were 0, 20, 100 and 1000 mGy and the animals were sacrificed at 1, 3 or 24 h post-irradiation. For long-term effects, the doses were 0, 50, 100, 250, 1000 and 2000 mGy and the animals were sacrificed after 1, 3 or 24 h or 10 months post-irradiation. X-irradiation and most post-mortem dissections were performed at Public Health England, Chilton. The eyes were surgically removed, fixed for 1–2 h in 4% (w/v) paraformaldehyde, washed and stored in sterile phosphate-buffered saline (PBS; 10 mM sodium phosphate, pH 7.4, 137 mM sodium chloride and 27 mM potassium chloride) and shipped the same day to Durham for analysis. This allowed the lens epithelium to be flat-mounted as described previously ([40] as modified by previous studies [10,41]). Briefly, with the use of a dissecting microscope and forceps, incisions on the lens posterior permitted the lens fibre mass to be gently removed. The lens capsule could then be flattened and pinned out onto a Sylguard silicone support, keeping the anterior hemisphere intact and with the lens epithelium exposed (figure 1). These preparations were then processed for immunofluorescence microscopy.

Murine lymphocyte isolation was done post-mortem, resulting in between 0.1 and 0.5 ml of whole blood collected by heart puncture. Blood was collected in EDTA tubes and immediately placed on ice to stop repair of DNA DSBs. The protocol was followed as described, with the exception that Histopaque density medium 1077 (Sigma-Aldrich Ltd, UK) was used for mouse lymphocytes [43].

To measure changes to the eye lens shape, 10 months after IR exposure eyes were dissected, lenses removed and put into M199 media (Gibco Life Technologies, UK) and images recorded for each lens (Nikon SMZ1500). Two measurements of the lens diameter at right angles were made, the ratio providing the aspect ratio for each lens. Cataract incidence in this strain of mice at 47 weeks is reported to be as high as 60% [44], making the observation of significant differences in the optical properties of lenses as a result of exposure to low-dose IR in such a small study impossible.

### 3.2. Irradiation of cultured FHL124 cell line

Human fetal lens epithelium FHL124 cell line [45] was maintained in DMEM supplemented with 10% (v/v) fetal calf serum (Sigma-Aldrich, UK) in a standard 5% (v/v) CO_2_ incubator on glass coverslips or plastic dishes until they reached 60–70% confluency. The cells were then exposed to IR in an X-ray irradiator at single doses of 0, 140, 280, 1130 or 2260 mGy (with doses varying from the previous experiments due to a necessary change in X-ray facility set-up in order to irradiate cells as opposed to live mice). One-hour post-irradiation, either cells were fixed in 4% (w/v) formaldehyde/PBS or proteins were extracted with Laemmli sample buffer [46] to produce processed total cell lysates.

### 3.3. Immunofluorescence microscopy analyses

The samples were permeabilized with 0.5% (w/v) Triton X-100 in PBS for 10 min and washed three times for 5 min in PBS. EdU incorporation was detected using an EdU Alexa Fluor488 Imaging Kit (Invitrogen, UK) according to the manufacturer's protocol. Primary antibodies: γH2AX (Millipore; 1 : 250); 53BP1 (Novus Biologicals; 1 : 250); RAD51 (Abcam; 1 : 250); MRE11 (Genetex; 1 : 250); TP53 (gift from Dr Borek Vojtesek (Moravian Biotechnology, Czech Republic)); cyclin D1 (Abcam; 1 : 250) were diluted in PBS/1% newborn calf serum (NCS) and applied overnight at 4°C. After removal of the primary antibodies and washing, samples were incubated for 1 h with the appropriate secondary antibodies (anti-mouse IgG TRITC (Sigma; 1 : 500) or anti-rabbit IgG (Sigma-Aldrich; 1 : 500)) with DAPI (4′,6-diamidino-2-phenylindole; Sigma; 1 : 1000) in PBS/1% NCS, and washed three times with PBS. An *in situ* cell-death detection kit, TMR red (Roche Diagnostics GmbH, Germany) was used to detect cell death in the lens epithelium. Coverslips were then mounted in glycerol/PBS (Citifluor Ltd, UK). The immunostained samples were imaged using an inverted microscope (Axioskop 40, Carl Zeiss Ltd, UK) with epifluorescence optics, images were collected and montages assembled in Adobe Photoshop v. 7.0/CS1.

### 3.4. Immunoblotting

FHL124 cell lysates were resolved by SDS PAGE (sodium dodecyl sulfate polyacrylamide gel electrophoresis) on 10% (w/v) polyacrylamide gels and transferred onto nitrocellulose membrane by the semi-dry blotting technique. The blots were incubated with glyceraldehyde 3-phosphate dehydrogenase (GAPDH) antibodies (loading control, Abcam, 1 : 1000); MRE11 (Genetex; 1 : 1000); TP53 (1 : 1000); anti-mouse IgG horseradish peroxidase (HRP; Stratech, 1 : 1000) or anti-rabbit IgG HRP (Stratech, 1 : 1000) and others listed above before being developed using ECL detection kit (GE Healthcare). For densitometry analysis, the bands were visualized using FUJIFILM IR LAS-1000 Pro v. 3.02 and relative densities measured using ImageJ. Three independent repeats were undertaken.

### 3.5. Statistical analyses

**Figure 2.**
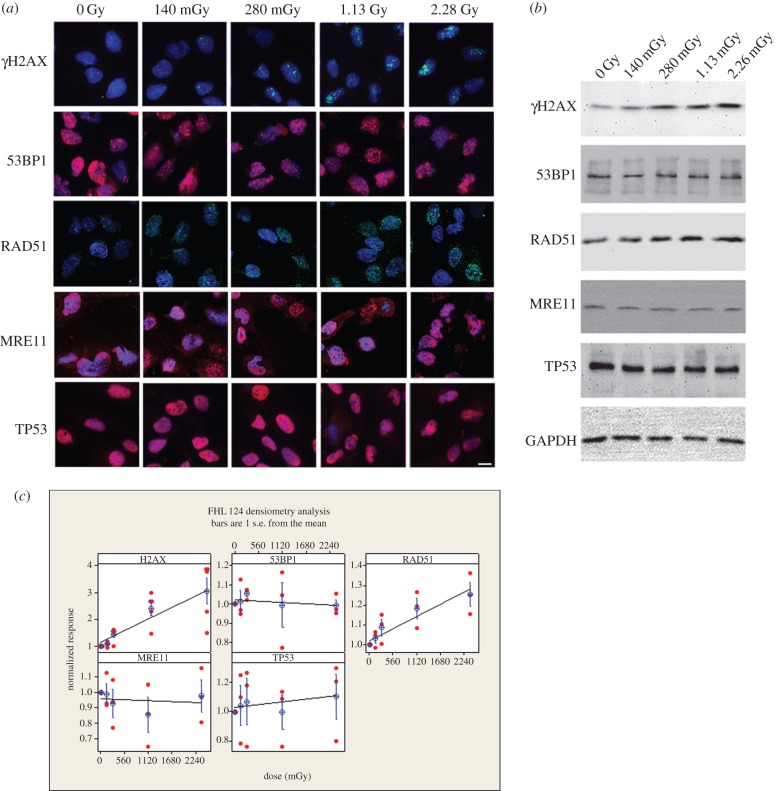
The susceptibility of human LECs to low-dose ionizing radiation. The human lens cell line FHL124 was exposed to low-dose IR up to 2.28 Gy. Exposed cells were then processed for both immunofluoresence microscopy (*a*) and immunoblotting (*b*) 1 h later. The signals obtained by immunoblotting were quantified and the mean from three independent experiments calculated and plotted (*c*) against IR dose. GAPDH was used as a loading control. Both γH2AX and RAD51 increased linearly with IR dose. Signals for other markers of DNA repair, 53BP1, MRE11 and TP53, remained unchanged as assessed by immunoblotting (*b*). By immunofluoresence microscopy (*a*), MRE11 and 53BP1 redistributed into nuclear foci, particularly at the 2.28 Gy level. TP53 remained uniformly distributed throughout the nuclear compartment, but excluded from nucleoli (*a*). As the levels of γH2AX and RAD51 increased after exposure to IR (*c*), so the number of nuclear foci also increased (*a*). Scale bar, 10 µm.

Almost all the datasets were normally distributed (Anderson Darling *p* > 0.05), which means Normal assumptions were appropriate for this data. Linear regression was applied to dose-response data, both with and without constants, with the *t*-test for significance of coefficients and analysis of variance (ANOVA) applied for significance of the overall fit. The general linear model (GLM) for ANOVA analysis with pairwise testing (Tukey's test) was used to assess the significance of the data in terms of the experimental factors. Apart from one dataset (see figure 2), there was no evidence of significant differences between experimental repeats. Different operators sometimes collected datasets from the same samples in order to check for operator bias (see figures 6 and 7). The different sets were formally assessed for differences before carrying out the full analysis. No evidence of any significant difference between operators was found (*p*-value always >0.05). Statistical analysis was carried out in Microsoft Excel^®^ and Minitab^®^ v. 15.

For the FHL124 human lens cell line, γH2AX, 53BP1, RAD51, MRE11 and TP53 band intensities were measured after exposure to 0–2260 mGy IR. Three independent repeats were made, the data from all three repeats forming the dataset for analysis. GLM ANOVA with pairwise testing (Tukey's test) was used to assess the significance of dose as well as to compare the repeats for each endpoint. For the γH2AX, RAD51 and 53BP1 foci in mouse lenses, GLM ANOVA was applied for the following factors: dose (levels: 0, 20, 100, 1000 mGy); time (levels: 1, 3 and 24 h); and zone (levels: central or peripheral); and interaction of factors was also investigated. Pairwise comparisons (Tukey's test) were applied for dose, time, dose × time and time × zone. For the analyses of cell density, EdU and cyclin D1 expression at 24 h post-irradiation, GLM ANOVA was applied for factors dose (0, 50, 100, 250, 1000 and 2000 mGy), zone (TZ or GZ), repeat, dose × region. Dunnett's test for comparisons with a control was used to assess the differences between dose levels, within regions where appropriate.

### 3.6. Nonlinear model development

We developed a novel statistical model to look for evidence that IR affected lens shape because the relation of changes in lens aspect ratio with IR dose did not appear to be linear, nor did the variation in aspect ratios appear to be normally distributed. Distortion of the lens aspect ratio was quantified as *y* = *w*_1_/*w*_0_ − 1, where *w*_1_ is the largest diameter measurement of the lens and *w*_0_ is the perpendicular measurement. Thus, *y* ≥ 0 and *y* = 0 indicates a non-distorted, circular lens. Mean lens distortion when exposed to radiation dosage, *x*, was assumed to be potentially nonlinear,3.1
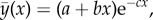
where *a*, *b* and *c* are constants. Setting *b* = *c* = 0 describes the case where distortion is independent of dosage, and setting *c* = 0 describes the case where distortion is linearly related to dosage.

Let *y_ij_* denote the distortion of lens *j* from mouse *i* (*j* = left (L) or right (R) eye), and let *x_i_* denote the associated radiation dosage. Variation in these distortion measurements showed a positive skew consistent with the exponential distribution (see figure 8*d*). Repeated measurements on mouse lenses were accounted for by assuming that variation in mean distortion between mice could be described by a gamma distribution. Given these assumptions, the likelihood of the model describing variation in the data, given all the distortion measurements, is3.2
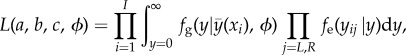
where *I* = 22 is the number of mice sampled, and *f*_g_ and *f*_e_ are the probability density functions of the gamma and exponential distributions, respectively. These functions are given by3.3
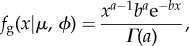
where *a*/*b* is the mean and *a*/*b*^2^ is the variance of the gamma distribution, and3.4
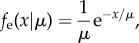
where *μ* is the mean and *μ*^2^ is the variance of the exponential distribution.

Likelihood ratio tests (LRTs) were used to seek statistical evidence that radiation dosage affected eye distortion, and whether any effect was linear or nonlinear. Specifically, linear effects were investigated by comparing the model having *c* = 0, denoted M (linear), with the model having *b* = *c* = 0, denoted M (null). Similarly, nonlinear effects were investigated by comparing the model having all parameters free, denoted M (nonlinear), with model M (null).

## 4. Results

### 4.1. Sensitivity of lens epithelium to low-dose ionizing radiation

For the initial studies of the lens response to low-dose IR, we selected the FHL124 human lens epithelium cell line as it shares 99.5% gene homology with native lens tissue and expresses phenotypic LEC markers [47]. Only low levels of γH2AX and RAD51 were detected in unexposed cultures and the cells responded in a dose-dependent manner to IR (within the 140–2280 mGy range tested) with the formation of nuclear γH2AX, 53BP1, RAD51 and MRE11 foci, as a result of DNA damage repair pathways being activated (figure 2). Semi-quantitative immunoblotting analysis confirmed the upregulation of γH2AX and RAD51 protein expression and the linear DNA damage response observed was statistically significant for both γH2AX and RAD51 (ANOVA *p* = 0.045 and <0.001, respectively), although post hoc testing indicated significant differences (*p* < 0.05) only between 0 and >1.13 Gy in both cases—possibly due to the small sample sizes employed here. For 53BP1, MRE11 and TP53, no significant dose-response was observed and no significant change in their levels of expression (figure 2*b*,*c*), but the proteins re-localized to form foci in the nuclei of the irradiated cells (figure 2*a*, 53BP1 and MRE11). Importantly, these data show that human LECs respond to low-dose IR as confirmed by changes in protein levels and the nuclear re-distribution of the markers of DNA damage.

**Figure 3.**
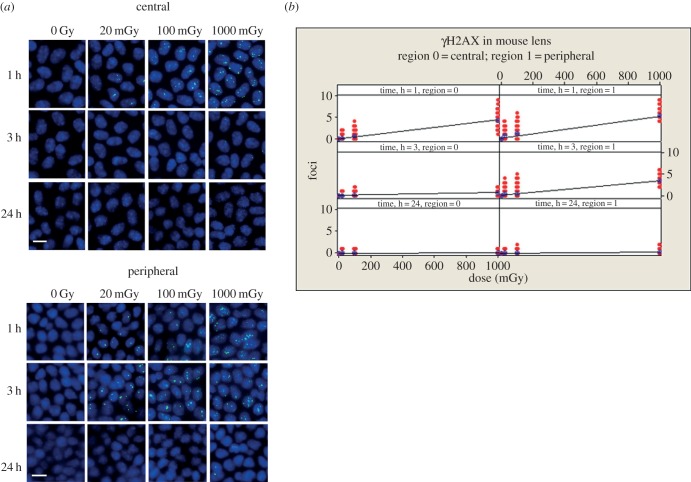
Dose-dependent increase in γH2AX foci in the nuclei of LECs after exposure to low-dose IR. Mice were irradiated with increasing levels of IR. At 1, 3 and 24 h, animals were sacrificed, the eyes removed and the lens dissected to remove the capsule and the attached LECs, which then was flat mounted prior to staining with antibodies to γH2AX. Representative images are shown for central and peripheral regions of the lens (*a*). The number of foci in the nuclei of LECs in the central and peripheral regions were then counted at the different time points and plotted with respect to dose (*b*). At the 1 and 3 h time points, the number of foci observed was dose dependent and linear regression demonstrated significant relationships with dose. GLM ANOVA revealed significant effects of dose, time and zone (*p* all <0.001) together with significant interaction effects between the factors (*p* ≤ 0.001). Scale bars, 10 µm.

To extend these findings still further, we investigated the response of the lens epithelium itself by exposing mice to a range (20–2000 mGy) of IR doses. LECs in culture have lost the spatial cues that typify the lens epithelium, where cell proliferation varies considerably dependent upon the location of the cells in the lens epithelium [6,48]. The ability to flat mount the lens epithelium following IR exposure represents a significant advantage for accurately counting nuclear foci, comparable to counting γH2AX in isolated blood lymphocytes. This is because the LECs are maintained as a cell monolayer that is attached to its own matrix, the lens capsule. In the first set of experiments, the early response (1–3 h) to low-dose radiation in the range 20–1000 mGy was studied, which is the time period when the majority of induced DNA damage should be actively repaired [33,49]. In mouse lens epithelia, even very low IR doses (20 mGy) were sufficient to stimulate the formation of γH2AX foci in both the central and peripheral regions (Tukey's pairwise *p*, 0 mGy versus 20 mGy, <0.001; figure 3). γH2AX foci persisted significantly (*p* < 0.001) longer in the peripheral (GZ and TZ) region of the lens compared with the central region where γH2AX foci have all but disappeared after 3 h. γH2AX foci caused by IR damage were no longer visible at the 24 h time points in all regions of the lens.

**Figure 4.**
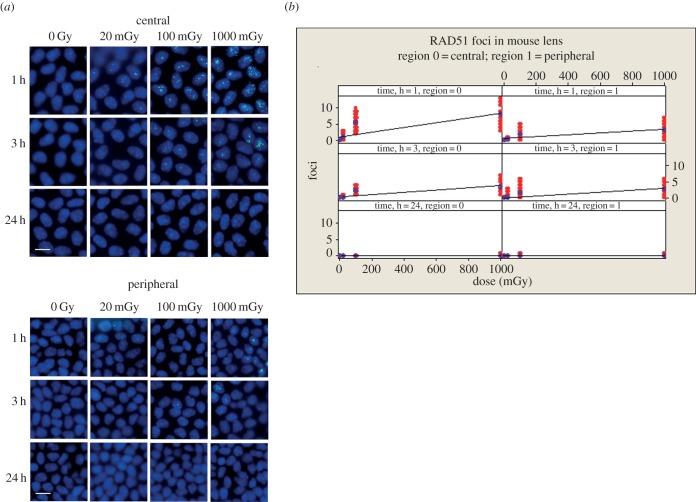
Formation of RAD51 containing foci in nuclei of LECs after exposure to low-dose IR. After irradiation (see figure 3 for detail), lenses were removed and flat mounted prior to staining with antibodies to RAD51 (*a*). RAD51 foci were readily detected in the nuclei of cells in both central and peripheral regions of the lens epithelium and the number seen were dose dependent at the 1 and 3 h timepoints (*b*). *T*-test *p*-value for the coefficients of the regression fits were all <0.001; ANOVA *p* for dose <0.001. Zone and time were also highly statistically significant (ANOVA *p* both <0.001). By 24 h, all RAD51 foci had disappeared. This time there was a significantly higher response in the central zone at 1 h (pairwise comparison for zones at 1 h, *p* < 0.001), but no difference between the zones at 3 h (pairwise comparison for zones at 3 h, *p* = 0.849). Scale bars, 10 µm.

The effect of IR on RAD51 was then investigated (figure 4). Significant differences between the central and peripheral regions of the mouse lens for γH2AX were also apparent for RAD51 (figure 4). Both the central and peripheral regions of the lens epithelium showed a significant dose-dependent increase in RAD51 foci after 1 h (*p* < 0.001), with post hoc testing demonstrating significant differences between all dose levels (*p* all ≤0.001) at 3 h in both the central and peripheral regions. These foci had disappeared 24 h post-irradiation (figure 4). In contrast to counts of γH2AX foci however, RAD51 foci were increased significantly (*p* < 0.001) in the central region compared with the peripheral region (figure 4).

**Figure 5.**
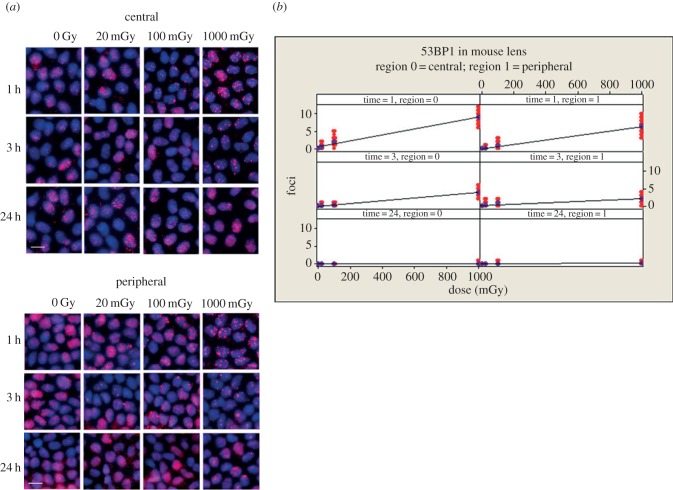
Dose dependent increase in 53BP1 foci in the nuclei of LECs after exposure to low-dose IR. After irradiation (see figure 3 for details), lenses were removed and flat mounted prior to staining with antibodies to 53BP1; representative images from the peripheral and central zones are shown (*a*). As expected, 53BP1 was readily detected in the nuclei of unexposed lenses, but the signal was concentrated into foci after IR exposure. *T*-test *p*-value for the coefficients of the regression fits of 1 and 3 h datasets were all <0.001; ANOVA *p* for dose <0.001. Zone and time were also highly statistically significant (ANOVA *p* both <0.001). The peripheral zone at 1 and 3 h (*p*-values responded significantly higher for pairwise comparisons for zone × time at 1 and 3 h both <0.001). By 24 h, the number of 53BP1 foci had returned to non-irradiated levels. Scale bars, 10 µm.

A similar analysis of 53BP1 was then performed (figure 5). Once again, there was a significant (*p* < 0.001) linear dose response for this marker of DNA repair of DSBs and a significant difference between all dose levels, including 0 and 20 mGy (*p* all < 0.001) at 3 h in both regions. By 24 h, the number of 53BP1 foci had returned to non-irradiated levels; however, the formation of large nuclear foci in the peripheral region was observed, particularly at 1 Gy (figure 5 bottom panel, arrows). These data counter the somewhat equivocal data obtained with the 53BP1 marker in the human cell line FHL124 (figure 2) and illustrate the complementarity of these mouse-based studies.

**Figure 6.**
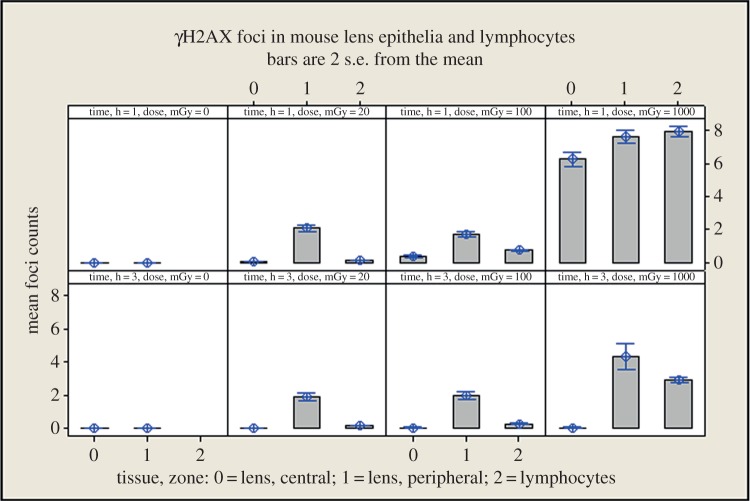
A direct comparison of γH2AX foci in mouse lymphocytes and central and peripheral LECs. Foci were counted for each of the indicated dose points at the 1 (top panels) and 3 h (bottom panels) time points.

In order to determine the relative radiosensitivity of the peripheral region to other cells in the irradiated mouse, we carried out a direct comparison of radiation-induced γH2AX foci in the mouse lens epithelium and circulating blood lymphocytes (table 1 and figure 6). The 24 h data are not shown as all responses at this time point were at baseline. These data demonstrate that both the central and peripheral regions of the mouse lens epithelium were significantly (*p* < 0.001) less sensitive to 1000 mGy compared with circulating blood lymphocytes. Cells in the central region of the lens epithelium appeared to repair DNA damage faster (figure 6; see 1000 mGy samples and cf. 1 and 3 h), but these were also not as sensitive (*p* < 0.003) as circulating blood lymphocytes across the whole dose range we tested. The peripheral zone was, in striking contrast, significantly (*p* < 0.001) more sensitive at both 20 and 100 mGy. Epithelial cells in the peripheral region of the mouse lens were therefore generally more sensitive to low-dose IR, as indicated by the number of γH2AX foci, than cells from the central region and peripheral blood lymphocytes from the same IR-exposed animals. These data identify for the first time regional nonlinear differences for the lens epithelium to low-dose IR (20 and 100 mGy).

**Table 1.** Statistical analysis of the effects of low-dose IR on LECs compared with circulating blood lymphocytes.

dose (mGy)	comparison	outcome	ANOVA *p* (pairwise comparisons)
20	central vs lymphocytes	central zone less sensitive than lymphocytes	<0.001
	peripheral vs lymphocytes	peripheral zone more sensitive than lymphocytes	<0.001
100	central vs lymphocytes	central zone less sensitive than lymphocytes	0.003
	peripheral vs lymphocytes	peripheral zone more sensitive than lymphocytes	<0.001
1000	central vs lymphocytes	central zone less sensitive than lymphocytes	0.003
	peripheral vs lymphocytes	peripheral zone less sensitive than lymphocytes	<0.001

### 4.2. Long-term effects of low-dose IR on lens growth

The formation of new lens fibre cells is entirely dependent upon cell proliferation in the GZ [50]. Altering the proliferation rate in the lens epithelium alters lens size and shape [7,8,10,11]. Preventing cell proliferation affords radioprotection to the lens [17,51]. With these points in mind, we considered the possible cellular consequences of the initial slower repair of DNA damage for the lens and its subsequent growth after exposure to low-dose IR. The ‘peripheral’ region was now analysed as two areas to consider potential differences between TZ (area 1) and GZ (area 2) that it contains.

**Figure 7.**
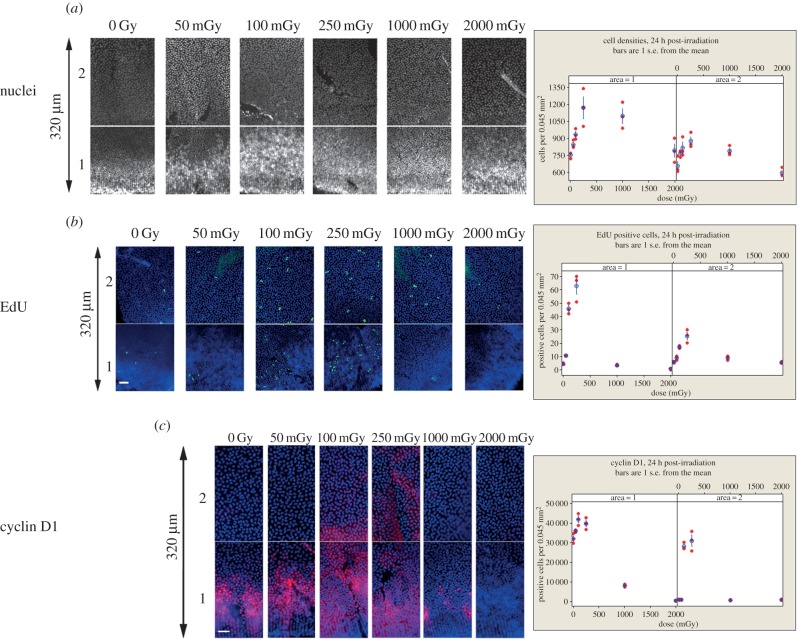
Effect of low-dose IR upon cell proliferation in the mouse lens epithelium. (*a*) Cell densities were measured in the peripheral region, for area 1 and area 2, 24 h after exposure to the indicated IR doses. Areas 1 and 2 are consecutive fields of view, separated by a few pixels, of the lens periphery. Both dose and area were significant factors (GLM ANOVA, *p* both <0.001). There was no significant interaction detected (area × dose *p* = 0.066). Dunnett's test for comparison with a control revealed that 250 and 1000 mGy both produced statistically significantly higher densities for area 1 (*p* = 0.002 and 0.007, respectively) and 100 and 250 mGy were significantly higher in area 2 (*p* = 0.042 and 0.007, respectively); by contrast, 50, 100 and 2000 mGy were statistically indistinguishable from the control (*p* all >0.05). (*b*) Cell proliferation was measured by EdU incorporation. Both dose and area were significant (GLM ANOVA, *p* both <0.001). There was a significant interaction between area and dose (*p* < 0.001); Dunnett's test for comparison with a control revealed that 100 and 250 mGy produced significantly higher EdU labelling (area 1, *p* both <0.001), a trend that was also observed in area 2, but with small differences between the labelling (*p* = 0.027 and <0.001, respectively). TUNEL staining showed no increase after IR exposure (data not shown). (*c*) Effects of IR upon cyclin D1 levels in the peripheral region of the lens. In controls, area 1 contains cells that are cyclin D1 positive, while area 2 does not. IR increased the cyclin D1 signal in area 2 for 100 and 250 mGy levels. Both dose and area were significant (GLM ANOVA, *p* both <0.001). A significant interaction between area and dose (*p* < 0.001) was observed. Dunnett's test for comparison with a control revealed that 100 and 250 mGy produced significantly increased cyclin D1 for area 1 (*p* < 0.001 and 0.005, respectively), whereas 1000 and 2000 significantly decreased the cell proliferation (*p* both <0.001); 50 mGy was not significantly different from the control. For area 2, only the 100 and 250 mGy points showed significantly higher levels of cyclin D1 (*p* < 0.001 in both cases); 50, 1000 and 2000 mGy were indistinguishable from the control (*p* all > 0.999). Vertical arrows, 320 µm. Scale bars, 25 µm.

Initial analysis of cell density (figure 7*a*) and EdU incorporation (figure 7*b*) performed 24 h following low-dose IR exposure demonstrated significant differential responses between the two areas in the peripheral region, which were not observed in the central region (data not shown). Doses of 100 and 250 mGy resulted in increased cell densities, though only significantly for 250 mGy (*p* = 0.15; *p* < 0.001, respectively) and EdU (both *p* < 0.001) incorporation in area 1. In area 2, the same trend was observed, albeit with smaller differences (figure 7*b*). The intensity of EdU labelling was not used to distinguish the progeny of labelled cells. Area 1 contains the TZ, whereas area 2 contains the GZ of the peripheral region of the lens. Again for 100 and 250 mGy, Cyclin D1 levels were also significantly increased in area 1 of the peripheral region (figure 7*c*, area 2), but after irradiation at higher doses (1000 and 2000 mGy they were also significantly reduced in the whole peripheral region (figure 7*c*, area 1 and 2). These data suggest that after exposure to low IR doses, LECs in the lens periphery re-enter the cell cycle, resulting in increased cell density in the peripheral region.

**Figure 8.**
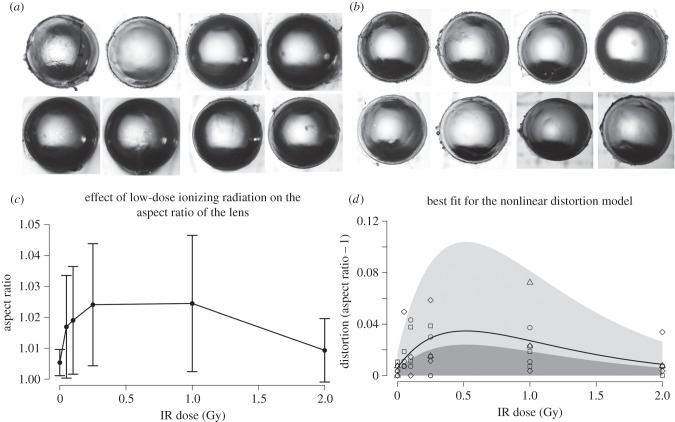
Long-term effect upon lens growth of IR exposure. The aspect ratio was measured and the datasets for control (*a*) and 1000 mGy (*b*) groups provided. Pairs of lenses from the same animal are displayed from left to right (*a*,*b*). When the axial ratio was plotted against IR dose (*c*), both the ratio and sample variance was increased for the treated lenses compared with those in the control group. In (*c*), error bars represent standard errors. Given we have observed nonlinear effects at the low-dose range and because there is increased variance in the sample data, we tested a best-fit nonlinear model. This was able to accurately predict the observed variation in the data (*d*). The nonlinear model described the data much better when compared with the null model (LRT, G2 = 11.07, *p* = 0.004), whereas a linear model was not the most appropriate model for the data relative to the null (LRT, G1 = 0.28, *p* = 0.598). Full details of the model and its development are in the Material and methods section.

We considered next whether these changes in cell density, cell proliferation and cyclin D1 expression would have longer term consequences for the lens itself by, for instance, affecting its shape. Therefore, we measured the aspect ratios of lenses 10 months after the initial exposure to IR (figure 8). Image datasets for control (figure 8*a*) and 1000 mGy exposed lenses (figure 8*b*) are shown. For a perfectly symmetrical lens, an aspect ratio of 1.0 would be expected (figure 8*c*) or zero distortion (figure 8*d*). For control lenses, this was measured as 1.0076 ± 0.0055. After exposure to 1000 mGy, the measured aspect ratio for the isolated lenses was 1.0245 ± 0.0221. A plot of aspect ratio versus IR dose (figure 8*c*) showed increased ratios and, most strikingly, increased variance for the exposed lenses. LRTs were used to seek statistical evidence that IR dose affected the mean aspect ratio and whether any effect was linear or nonlinear. The nonlinear model much better described the data when compared with the null model (LRT, *G*_2_ = 11.07, *p* = 0.004), whereas the linear model was no better at describing the data relative to the null (LRT, *G*_1_ = 0.28, *p* = 0.598). These two tests support the nonlinear model as the best descriptor of the data (figure 8*d*). Importantly, our assumption that variation in aspect ratios had an exponential distribution was supported by the standard deviation of aspect ratios being well approximated by the mean (figure 8*d*). Our models gave very similar fits when we set *ϕ* = 0, indicating that animals varied little in their susceptibility to distortion (e.g. there was no evidence that some individuals were relatively unaffected by radiation); we found that intermediate levels of radiation resulted in the highest variation in aspect ratio measurements between eyes of the same individual. The long-term consequences of low-dose IR exposure resulted in significant distortion in the lens aspect ratio. However, such distortions were not as frequently observed at higher (2000 mGy) doses, where cell proliferation was halted rather than stimulated (figures 7*c* and 8*d*). These data again support the conclusion that there are nonlinear, stochastic biological responses by the epithelial cells in the lens periphery at low doses of IR, which differ from the responses at higher doses.

## 5. Discussion

### 5.1. Low-dose ionizing radiation induces nonlinear biological responses in the epithelial cells in the lens periphery

The major finding of this study was the demonstration that IR has distinct effects dependent upon both the regions of the lens epithelium being investigated and the doses to which they are exposed. We have demonstrated DNA DSB responses (figure 3) and repair reliant on both HR (RAD51; figure 4) and NHEJ (53BP1; figure 5) were activated in response to IR in the mouse lens epithelium across a range of doses. The most significant findings, however, were that DSB repair after low-dose IR (20–100 mGy) compared with higher dose (1000 mGy) was delayed as monitored by the persistence of γH2AX foci in the peripheral region of the mouse lens (figures 3 and 6), was coincident with increased cell proliferation and increased cell density in the lens periphery (figure 7) and produced statistically significant lens shape changes (figure 8). Moreover, we have also established that at low IR doses (20 and 100 mGy), the peripheral region of the lens was more sensitive than either the central region or peripheral blood lymphocytes (figure 6 and table 1). Although these dose points are most relevant to the current ICRP recommendation [22], further dose points within the 100–1000 mGy range would help compel this point.

Our data concerning the persistence of γH2AX foci in the periphery of the irradiated mouse lens (figures 3 and 6) suggest that the repair of DSBs is slower than either cells in the central region of the mouse lens epithelium or in circulating blood lymphocytes (figure 6 and table 1). In the mouse lens, those cells that are actively replicating their DNA, i.e. those in the GZ within the peripheral region of the lens epithelium, are more at risk because the complementary DNA strands in the duplex will be separated at this time, increasing the probability that the ends of the DSBs are joined incorrectly. Although this study was predominantly focused on early lens changes in response to IR, we believe this could be a major contributory factor in the appearance of lens shape abnormalities 10 months later.

### 5.2. Sensitivity of lens epithelial cells in tissue culture compared to the lens

Using primary human [52] and mouse LECs [53], others have reported a linear dose-response to low-dose IR. The data presented here extend these previous studies in terms of the use of an established human LEC line (FHL124) alongside additional markers and by providing additional evidence at low doses. Lens cells in tissue culture do not follow completely the situation in the eye lens as tissue culture induces a normalization of size and growth characteristics not seen when cells are first isolated from the lens epithelium [53]. Most cells in the lens epithelium are usually arrested in G1 of the cell cycle [54]. In the context of tissue culture based studies, such spatial distinctions that define the lens epithelium [6,55] are lost when these cells are placed into tissue culture. We have demonstrated that the human cell line FHL124 showed linear dose-response curves for two (γH2AX and RAD51) of the five markers, while two others (53BP1 and MRE11) both redistributed into nuclear foci (figure 2). These data suggest that cultured human LECs respond similarly to low-dose IR compared with the mouse lens epithelium, but within the limitations afforded by tissue culture [53].

### 5.3. Nonlinear effects upon the lens epithelium

It is well established that cell proliferation ceases in the GZ of the lens epithelium after IR exposure (more than 15 Gy), causing a decrease in GZ cell density and the disorganization of cells in the TZ and MR within the peripheral region [16,56,57]. The effects on cell proliferation are probably due to IR-induced DNA damage causing TP53 stabilization, the induction of the CDK2 inhibitor p21 and the proteasomal degradation of cyclin D1 leading to G1/S phase cell cycle arrest [58,59]. Cyclin D1 is an important component of the cellular response to genotoxic stress as interference with its degradation can render cells more susceptible to DNA damage [60], its long-term elevation leading to genomic instability after protracted IR exposure [61]. Cyclin D1 is involved in DSB repair through an interaction with RAD51 [38,39]. It is also involved in the adaptive response of cells to low-dose IR exposure [62]. It is interesting to note that Hamada & Fujimichi [26] observed nonlinear dose responses for *in vitro* proliferation in a human LEC line, however proliferation was measured in terms of percentage of large colonies, the dose range was much larger (0–6 Gy) than in this study, and here proliferation was only measured in excised lenses, so the potential for detailed comparison is limited.

In this study, the observation that mouse LECs responded to higher doses of IR (1000–2000 mGy) by a reduction in EdU incorporation and a reduction in cyclin D1 levels in the GZ is consistent with cell cycle arrest 24 h post-irradiation in the GZ and mirrors observations made in other animal models using much higher (more than 9 Gy) IR doses [56,63,64]. It is also consistent with models where cell proliferation in the GZ has been compromised [11,40]. By contrast, low doses of IR (100–250 mGy) promoted EdU incorporation and increased cyclin D1 levels in the peripheral region, which is consistent with more cells being in the cell cycle and the increased cell densities 24 h following IR exposure. These data provide important evidence for nonlinear responses to low-dose IR in the lens periphery, i.e. GZ and TZ compared, with the central region as well as explaining cell cycle arrest caused by high IR doses. Clearly, the biological responses to high- and low-dose IR are quite different in terms of their cell cycle effects.

From studies of cataract incidence in astronauts, others have also concluded that low-dose IR can elicit such nonlinear biological responses in the lens [24]. This has also been reported for heart, small intestine, kidney and skin mouse tissues [49,65] and for human primary fibroblast cell lines [66]. Interestingly, in earlier studies on effects of high doses (more than 9 Gy) upon the rat eye lens, part of the irradiated lens was shielded and used to provide a baseline for cell proliferation. A dose of 340 mGy was calculated for the shielded portion when the whole lens was exposed to 9.6 Gy [64]. It is perhaps then not a coincidence that proliferation rates were also significantly increased in the shielded portion of those irradiated lenses [64]. These offer further support to the significant nonlinear biological responses to low-dose IR in the eye lens we have reported here.

### 5.4. Double strand break repair, cell proliferation and the effect of low-dose ionizing radiation on the eye lens

**Figure 9.**
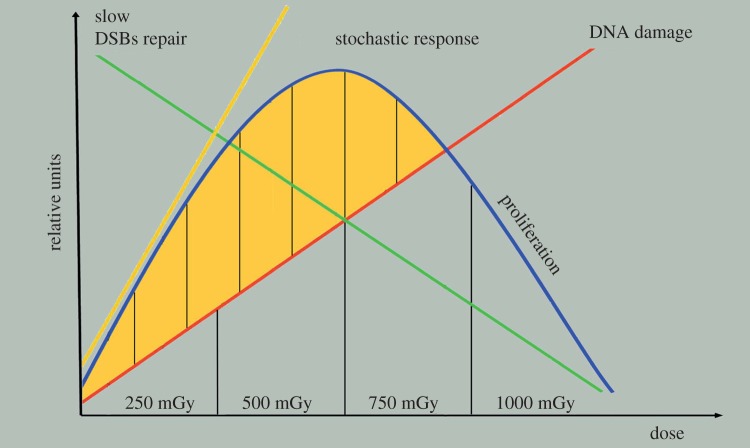
Working model based on the linear dose response of cumulative DNA damage with IR dose in lens epithelium. Low levels of DNA damage at low doses triggers slow responses to DSBs in the lens periphery and these delayed repair kinetics coincide with re-entry of these cells into the cell cycle. This coincides with increased levels of cyclin D1. Such responses are increased with IR dose, but limited by the cumulative DNA damage and incorporating the nonlinearity and stochastic nature of these responses. Cell proliferation in the peripheral region peaks within the range of 250–500 mGy.

A mechanistic link between low-dose IR and the deficiencies in DSB repair and the increased proliferative response and increased levels of cyclin D1 of the epithelial cells in the periphery of the lens epithelium as observed in this study is plausible (figure 9). Cumulative DNA damage occurs with IR dose, but at low-dose IR this DNA damage triggers a retarded response to DSBs resulting in delayed repair kinetics that is accompanied by re-entry into the cell cycle, evidenced by the elevated levels of cyclin D1 and increased cell density. It has been previously established that DSBs labelled by γH2AX caused by very low IR doses (1 mGy) are not as efficiently repaired as those sustained at higher IR doses [49,67]. Low (10 mGy) dose IR exposure also produces different transcriptional profiles compared with higher (more than 200 mGy) doses [49]. Such stochastic biological responses will be likely to increase with IR dose, but then they become limited by the cumulative DNA damage because this is linear with dose. In our schematic, cell proliferation peaks at about 500 mGy (figure 9) based on the best-fit nonlinear model of the lens aspect ratio data (figure 8*d*). This increase in cell proliferation at low IR doses will consequently preserve the irreversible changes associated with DNA damage, which would be expected to compromise the proliferative potential of these cells at the time of IR exposure (figure 7). At very high doses (15 Gy), it has long been known that in the first hours and first 3–4 days after IR exposure, all mitotic activity ceases [16], followed after by a short period (one week) of increased cell division before returning to pre-exposure levels [16]. We interpret these data to indicate that DNA repair is completed before any cell division is resumed in the rabbit lens epithelium. Interestingly, the accumulation of the large 53BP1 foci in the nuclei of LECs in the peripheral region (figure 5) is a feature that is commonly associated with a persistent DNA damage response and telomere-initiated senescence in mammalian cells [68,69].

## 6. Conclusion

The results of this study have demonstrated that: (i) peripheral LECs repair DSBs after exposure to 20 and 100 mGy more slowly than circulating blood lymphocytes; (ii) initial effects within 1–3 h of IR exposure appear to follow linear-no-threshold responses at low doses; however (iii) later time points (24 h–10 months) revealed nonlinear biological responses, with evidence of differential low (less than 1000 mGy) and high dose-responses.

Ideally, the interpretation of current epidemiological data [18–21,25,70,71] should reflect these observations and the data presented in this study. As cataracts have long been assumed, however, to be a deterministic effect of radiation exposure with a threshold on the order of 2 Gy for acute exposures, and because accurate individual low-dose dosimetry is not easy, there is very little high-quality epidemiological data currently available at low doses. The ICRP's proposals for lower occupational dose limits have now been incorporated into the revised EU BSS [23], which represents a legal requirement for EU countries, so it is very important to now firmly establish the mechanisms of IR induced cataract at low (less than 0.5 Gy) doses. It has been demonstrated here that low-dose IR effects on the GZ and TZ in the peripheral region of the lens are due to a combination of biological responses that include less-efficient DNA repair at the same time as cell proliferation is increased (figure 9). This combination of events lends support to the previous suggestion that low and high doses of IR cause different effects in LECs [24] and this offers an explanation why the increased passage of time (i.e. follow-up in long-term epidemiological studies) has led to the observation of effects at lower doses than previously [22]. In addition, the data in this study can be interpreted to further suggest stochastic processes can explain the biological responses of epithelial cells in the lens periphery (figure 9). The next stage in the investigation will be to determine how DNA damage, DNA repair, cell proliferation and cell differentiation interact to affect the cellular response of lens cells and their different timescales.

## Data accessibility

Primary data from this study concerning foci, nuclei and cell counts, lens measurements and band and immunofluorescence signal intensities will be deposited in the DRYAD Digital Repository (http://datadryad.org). All statistical methods are detailed in the Material and methods section.
